# Transcriptional and Post-Transcriptional Regulation of Autophagy

**DOI:** 10.3390/cells11030441

**Published:** 2022-01-27

**Authors:** Qiuqin Ma, Shihui Long, Zhending Gan, Gianluca Tettamanti, Kang Li, Ling Tian

**Affiliations:** 1 Guangdong Provincial Key Laboratory of Agro-animal Genomics and Molecular Breeding, College of Animal Science, South China Agricultural University, Guangzhou 510642, China; 20172026002@stu.scau.edu.cn (Q.M.); 20201025002@stu.scau.edu.cn (Z.G.); 2 Guangdong Laboratory for Lingnan Modern Agriculture, Guangzhou 510642, China; 3 Guangdong Provincial Sericulture and Mulberry Engineering Research Center, College of Animal Science, South China Agricultural University, Guangzhou 510642, China; 4 Guangdong Provincial Key Laboratory of Insect Developmental Biology and Applied Technology, Institute of Insect Science and Technology, School of Life Sciences, South China Normal University, Guangzhou 510631, China; 20132500059@m.scnu.edu.cn; 5 Department of Biotechnology and Life Sciences, University of Insubria, 21100 Varese, Italy; gianluca.tettamanti@uninsubria.it; 6 BAT Center-Interuniversity Center for Studies on Bioinspired Agro-Environmental Technology, University of Napoli Federico II, 80138 Napoli, Italy

**Keywords:** autophagy, regulatory mechanisms, transcription, ncRNA, RNA methylation

## Abstract

Autophagy is a widely conserved process in eukaryotes that is involved in a series of physiological and pathological events, including development, immunity, neurodegenerative disease, and tumorigenesis. It is regulated by nutrient deprivation, energy stress, and other unfavorable conditions through multiple pathways. In general, autophagy is synergistically governed at the RNA and protein levels. The upstream transcription factors trigger or inhibit the expression of autophagy- or lysosome-related genes to facilitate or reduce autophagy. Moreover, a significant number of non-coding RNAs (microRNA, circRNA, and lncRNA) are reported to participate in autophagy regulation. Finally, post-transcriptional modifications, such as RNA methylation, play a key role in controlling autophagy occurrence. In this review, we summarize the progress on autophagy research regarding transcriptional regulation, which will provide the foundations and directions for future studies on this self-eating process.

## 1. Introduction

Macroautophagy, hereafter referred to as autophagy, is the main type of autophagy, which is characterized by the formation of autophagosomes. Autophagosome biogenesis involves a series of autophagy-related (Atg) proteins that accompany the different steps of the autophagic process. Autophagosome initiation is mediated by the activity of the ULK1/Atg1-ATG13/Atg13 protein kinase complex. Nucleation of the autophagosome requires the BECN1/Beclin 1/ATG6-PIK3C3/Vps34 (catalytic subunit of the class III phosphatidylinositol 3-kinase (PtdIns3K)) complex. Elongation and maturation of the autophagosome involves two ubiquitin-like systems, i.e., Atg5–Atg12-Atg16 and LC3/Atg8–phosphatidylethanolamine (PE). Finally, the mature autophagosome fuses with the lysosome, leading to cargo degradation and completing the autophagic flux.

Autophagy is a well-regulated physiological process that is implicated in development, metabolism, immunity, neurodegenerative diseases, and tumorigenesis [1]. Induction of autophagy is accompanied by an increase in mRNA levels of certain *Atg* genes, *WIPI1* (WD repeat protein interacting with phosphoinositides), *p62/SQSTM1*, and *vacuolar (H+)-adenosine triphosphatases* (*V-ATPases*), or by a variation of post-translational modifications of autophagy-associated proteins [2,3,4,5]. Nutrient deprivation, endoplasmic reticulum (ER) stress, hypoxia, lipotoxicity, cholesterol, and insect molting hormone can all affect the transcription of *Atg* genes. In response to starvation and circadian signals, FXR (farnesoid X receptor) and PPARα (peroxisome proliferator-activated receptor alpha) oppositely regulate the great variation of *Ulk1* transcription in mammalian liver cells, accompanied by limited changes in mRNA levels of *GABARAPL1*, *Bnip3*, and *LC3b* [6]. ER stress and hypoxia increase the transcription of *ULK1/ULK2, Atg5*/*ATG5*, *ATG4B*, *ATG13*, *LC3*, and *GABARAPL1* in several mammalian cancer cells [5,7]. In mouse liver fibrosis, insulin-like growth factor-binding protein-related protein 1 (IGFBPrP1) increases the expression of *Atg9a*, which encodes the sole transmembrane protein and delivers membrane to the expanding phagophore, and thereby formation of the autophagosome [8,9]. On the other hand, the transcription of *ULK1* and *ULK2* is repressed by the chromatin non-histone protein HMGA1 (high-mobility-group AT-hook 1) during the initiation and progression of malignant neoplasia such as skin cancer [10]. In addition, the transcription of *V-ATPases* from the V0 and V1 subunits are consistently unregulated during the induction of autophagy by upstream signals to facilitate the flux [3,4,5]. Autophagy regulates the homeostasis of cholesterol, whereas cholesterol and its derivatives, such as the insect-molting hormone 20-hydroxyecdysone (20E) and 27-hydroxycholesterol, are able to induce autophagy by promoting the transcription of *Atg* genes, as well as inducing the deacetylation of ATG proteins in both *Bombyx mori* and mammals [11,12]. Here, we summarize the most recent studies on the regulation of autophagy at the mRNA level, and provide a deep thinking and prospects in studies on autophagy. 

## 2. Regulation of Autophagy by Transcriptional and Post-Transcriptional Modifications

### 2.1. Transcription Factors Regulate Autophagy at RNA Level

Several transcription factors play critical roles in regulating autophagy. The transcription factor EB (TFEB), a basic helix loop helix (b-HLH) leucine zipper protein from the microphthalmia-associated family (MiT/TFE), is one of the key transcription factors first identified to mediate autophagosome formation and autophagosome–lysosome fusion under starvation, in addition to its role in lysosomal biogenesis [13]. In mice liver, the circadian pattern of *Atg* gene expression depends on nutrient-sensitive activation of TFEB and TFE3: in the absence of nutrients (supplied with light), TFEB and TFE3 translocate to the nucleus and upregulate the expression of *Atg3*, *Atg5*, *Bnip3*, and *LC3*, which are involved in autophagy [14]. TFEB positively regulates the expression of genes involved in lysosomal biogenesis and autophagy during starvation in mouse liver, so autophagy shuttles lipid droplets to the lysosome for hydrolysis. Moreover, TFEB overexpression rescues obesity syndrome and lipid metabolism in *Atg7* liver-KO mice, in which autophagy is blocked and lipids accumulate in the liver. Thus, TFEB is proven to prevent diet-induced obesity in mice by mediating autophagy [15]. Interestingly, the zinc-finger-family DNA-binding protein (ZKSCAN3) inhibits starvation-induced autophagy, and knockdown of *ZKSCAN3* can promote TFEB-induced autophagy [16]. 

Members of FOXO (forkhead Box O) family can regulate autophagy induction at the transcriptional level. Notably, adenovirus-mediated expression of constitutively active *FOXO3* (*ca-FOXO3*) causes dramatic atrophy in mouse muscles and myotubes, since FOXO3 increases the autophagic flux by binding to the promoters of *LC3b*, *Atg12L*, and *Gabarapl1* and directly increasing their transcription [17,18]. In lung cancer cells, acetylated FOXO1 activates *ATG7* expression to enhance autophagy, and it is thus implicated in the suppression of tumor growth through autophagy activation [19]. AMPK (AMP-activated protein kinase) is activated by glucose starvation. Subsequently, activated AMPK phosphorylates FOXO3a and leads to its nuclear translocation, followed by the upregulation of *CARM1* (*co-activator-associated arginine methyltransferase 1*), which coactivates autophagy with TFEB by increasing the transcription of autophagy- and lysosome-related genes [20].

Nuclear receptors sensitive to metabolism play key roles in autophagy occurrence. PPARα is activated by fatty acids to promote their oxidation under starvation conditions, whereas FXR is activated by bile acids returning to the liver under nutrient-rich conditions. Consistently, PPARα is required for the full induction of autophagy by starvation, whereas FXR is needed for the suppression of autophagy in the liver of fed mice. PPARα and FXR competitively bind to shared sites in the promoters of autophagy-associated genes and control the expression of *Atg7*, *Beclin1*, *Bnip3*, and *LC3* [6]. Small heterodimer partner (SHP), which is an orphan nuclear receptor responsible for maintaining the homeostasis of bile acids, is required for hFGF19 (bile acid-induced fibroblast growth factor-19, mFGF15)-mediated inhibition of hepatic autophagy, and plays a negative role in autophagy induction through FGF19-SHP-LSD1 axis by repressing the expression of most autophagy-associated genes, including *Atg3*, *Atg5*, *Atg7*, *Atg10*, *WIPI1*, *Uvrag*, and *Tfeb*. [21,22]. In preadipocyte 3T3-L1 cells, adipogenic transcription factors C/EBPβ (CCAAT/enhancer binding protein beta) and PPARγ (peroxisome proliferator-activated receptor gamma) directly bind to the promoter region of autophagy genes, leading to the expression of *LC3*, *Beclin1*, and *Atg4b*, to facilitate autophagy. C/EBPβ and PPARγ directly bind to the promoters of *TFEB* and *FOXO1*, too, to indirectly control the expression of autophagy-associated genes [23]. The transcription factor E2F1 (E2 transcription factor 1) not only mediates apoptosis, but also enhances autophagy by binding to the promoters of *LC3, ATG1, ATG5*, and *DRAM* (*damage-regulated autophagy modulator*) to upregulate their expression, showing a positive role for E2F1 in DNA damage-induced autophagy [24]. In LNCaP and HeLa cells, the ER stressor tunicamycin induces the transcription of *ATG16L1, GABARAP, ATG12, ATG5, ATG3*, and *BECN1* to upregulate autophagy through the activation of ATF4 (transcription factor 4) [5]. Furthermore, *Atg* gene expression is also linked to the status of histone acetylation: the inhibition of histone deacetylase sirtuin1/2 increases the expression of *ATF4* to induce autophagy, playing a pro-survival role in human NSCLC (non-small cell lung cancer) cells [25].

In insects, several transcription factors have been documented to mediate autophagy. In *Drosophila melanogaster*, E93, a downstream transcription factor of 20E signaling induces both autophagy and caspase activity by blocking PI3K-MTORC1 signaling [26]. The transcription factor FOXO prevents the aggregation of damaged proteins by promoting the expression of *Atg1, Atg5, Atg6*, and *Atg8* in *D. melanogaster* [27]. Zika virus (ZIKV) triggers NF-κB-dependent inflammatory signaling in the fly brain and induces the expression of *Atg5* and *Atg7*, leading to autophagy activation in neurons and limiting the infection and proliferation of ZIKV in this organ [28]. In *B. mori*, 20E upregulates the downstream transcription factors *BmBr-C, BmE74, BmHR3*, and *Bmβ-ftz-F1* and thus determines the transcriptional induction of most of *Atg* genes to promote autophagy, which is essential for larval tissue remodeling during metamorphosis [11,29]. Recent studies have shown that 20E and starvation are both able to activate BmTFEB in *B. mori* to promote the transcription of *BmV-ATPases* and the assembly of the subunits, thus triggering lysosomal acidification and the autophagic flux [4]. Moreover, ACSS2 (acyl-CoA synthetase short-chain family member 2) forms a complex with TFEB, which facilitates the acetylation of histone using acetyl-CoA as an acetyl donor, and then promotes the transcription of TFEB-targeted genes in the nucleus, enhancing lysosomal biogenesis and autophagy [30].

Transcriptional regulation of autophagy is evolutionarily conserved between insects and mammals [12]. Notably, transcription factors have been reported to regulate autophagy in plants, too. In *Arabidopsis thaliana*, the transcription factor TGA9 (TGACG motif-binding protein 9) is confirmed to be a positive regulator of autophagy. The overexpression of *TGA9* upregulates the mRNA levels of *Atg* genes and induces autophagy [31]. Transcription factors and their function in the regulation of autophagy are listed in Table 1.

### 2.2. Regulation of Autophagy by Non-Coding RNAs

In addition to the transcription factors reported above, non-coding RNAs represent key regulators of autophagy. Non-coding RNAs mainly include microRNA, circRNA, and lncRNA. A series of non-coding RNAs are able to mediate the occurrence of human diseases and drug sensitivity in therapy by modulating autophagy [41]. MicroRNAs (miRNAs), about 22 nucleotides long, are conserved in evolution and expressed in almost all eukaryotes. Interestingly they have been identified as sequence-specific post-transcriptional regulators of gene expression, including *Atg* genes [42]. *miRNA-101* inhibits autophagy by targeting *RAB5A*, a member of the RAS oncogene family, and *ATG4d*, leading to the suppression of tumor formation [43]. Moreover, *miRNA-101* and *miRNA-376b* inhibit the expression of *ATG4c* and *ATG4d*, respectively [44]. Finally, *miRNA-103a-3p* directly targets *Atg5* to inhibit autophagy and protect cardiomyocytes [45]. In *Caenorhabditis elegans*, *miRNA-83* disrupts autophagy in multiple tissues by inhibiting *cup-5* (autophagy regulator), whereas *miRNA-34* inhibits the autophagic flux in vitro and affects the protein levels of Atg9, which is evolutionarily conserved in mammals [46,47]. In summary, according to the current literature, all microRNAs negatively regulate autophagy by directly inhibiting the expression of *Atg* genes, which are involved in the occurrence of diseases such as cancer and aging.

CircRNAs, formed by head-to-tail splicing of exons, are naturally generated from the family of non-coding RNAs, and show a regulatory role in gene expression at the post-transcriptional level [48,49]. In astrocytes, circRNA *NF1-419* upregulates the expression of *ULK1, BECLIN1, ATG5, ATG12*, and *ATG13* by binding to Dynamin-1 and adaptor protein 2 B1(AP2B1) [50], whereas circRNA *PABPN1* blocks the binding of human antigen R (HuR) to *ATG16L1* mRNA and thus inhibits autophagy in human intestinal epithelial cells [51]. HuR is reported to upregulate *ATG7*, *LC3II*, and *ATG16L1* expression to enhance autophagosome formation [52]. Thus, autophagy is differentially regulated by multiple circRNAs.

In mammals, genomic transcription produces a large number of long non-coding RNAs (lncRNA), which can regulate *Atg* genes expression and thus mediate autophagy occurrence [42]. In mouse, *lncRNA NEAT1* directly binds to *miR-29b* and then upregulates *Atg9a* expression to activate autophagy; similarly, *LncRNAXIST* enhances ethanol-induced autophagy by binding to *miRNA-29b* [8,53]. In human gallbladder cancer tissues, *lncRNA GBCDRlnc1* increases the expression of phosphoglycerate kinase 1 (PGK1), which upregulates *ATG5* and *ATG12* expression. Moreover, PGK1 phosphorylates BECLIN1 to induce autophagy [54,55]. Antisense intronic l lncRNA *eosinophil granule ontogeny transcript* (*Ai-lncRNAEGOT*) enhances autophagosome formation, as well as paclitaxel sensitivity in human cancer [56]. However, *lncRNA HOX transcript antisense RNA (HOTAIR)* downregulates the expression of *LC3B, BECLIN1, ATG3*, and *ATG7* to inhibit autophagy, which suppresses the invasion of oral squamous cell carcinoma cells [57]. Non-coding RNAs participating in autophagy are listed in Table 2.

### 2.3. Regulation of Autophagy by RNA Methylation

**Figure 1:**
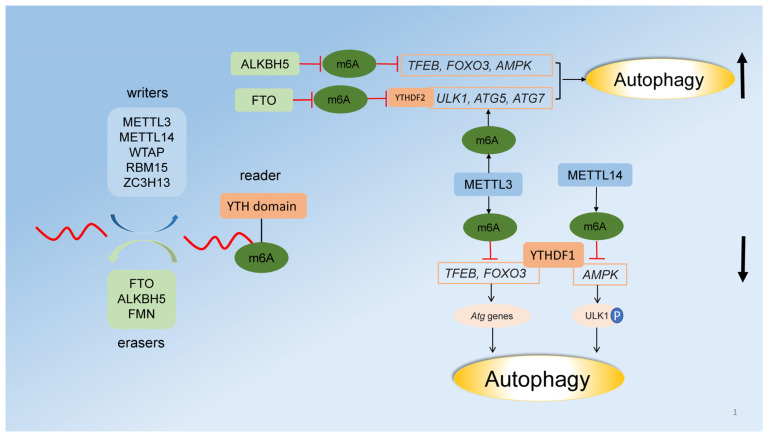
Schematic diagram of m^6^A modification and its regulation of autophagy. m^6^A modification is mediated by the methyltransferases (writers) METTL3, METTL14, WTAP, RBM15, and ZC3H13 and the demethylases (erasers) FTO, FMN, and ALKBH5. m^6^A indirectly affects RNA processing by recruiting reader proteins, which contain the YTH domain [79,80]. FTO upregulates *UKL1*, *ATG5*, and *ATG7* expression to induce autophagy by YTHDF2-dependent targeting of their mRNA. ALKBH5 demethylates *TFEB*, *FOXO*, and *AMPK* mRNAs to activate autophagy. *FOXO*, *TFEB*, and *AMPK* are the targets of m^6^A reader protein YTHDF1. METTL3 increases m^6^A levels of *UKL1*, *ATG5*, and *ATG7* to upregulate autophagy, whereas METTL3 and METTL14 negatively regulate autophagy through m^6^A methylation of *TFEB*, *FOXO*, or *AMPK* mRNAs, which are responsible for the expression of *Atg* genes or ULK1 phosphorylation. P indicates phosphorylation.

N6-methyl-adenosine (m^6^A) modification of mRNAs is pervasive and highly conserved in eukaryotic cells. m^6^A modification is mediated by methyltransferases (writers) consisting of methyltransferase-like 3 (METTL3), METTL14, Wilms tumor 1-associated protein (WTAP), RNA-binding motif protein 15 (RBM15), and zinc-finger CCCH domain-containing protein 13 (ZC3H13) [79]. The demethylases (erasers) reported in m^6^A modification are represented by fat mass and obesity-associated protein (FTO), flavin mononucleotide (FMN), and α-ketoglutarate-dependent dioxygenase alkB homolog 5 (ALKBH5) [80]. m^6^A modification also indirectly affects RNA processing by recruiting reader proteins, which harbor the YT521-B homology (YTH) domain (Figure 1) [79].

METTL3 positively regulates autophagy by increasing the expression of *ATG5*, *ATG7*, and *LC3B* through m^6^A modification of their mRNA, whereas β-elemene reverses gefitinib resistance in gefitinib-resistant PC9GR and HCC827GR derived from NSCLC cells by inhibiting METTL3-mediated autophagy [81]. Moreover, METTL3 suppresses autophagy by methylating *FOXO3* (in an 800 bp region of *FOXO3* 3′UTR containing the m^6^A modification site), which subsequently downregulates the expression of *ULK1*, *ATG5*, *ATG7, ATG12, ATG16L1*, and *MAP1LC3B* in human sorafenib-resistant hepatocellular carcinoma [82]. Similarly, METTL3 methylates the 3’ UTR of *TFEB* mRNA and thus inhibits autophagy [83].

Demethylase FTO increases the autophagic flux in patients with chronic kidney disease [84,85]. In particular, FTO upregulates *ULK1* expression by demethylating the adenine residues 3335, 3397, and 3784 at 3’ UTR of *ULK1*, thus promoting autophagy in Hela cells. In a mouse preadipose cell line, mRNAs of *Atg5* and *Atg7* are the targets of m^6^A reader protein YTHDF2, and FTO upregulates *Atg5* and *Atg7* expression in a YTHDF2-mediated manner to promote autophagy [86]. There is a close interaction between demethylases or methyltransferases and the autophagic pathway. In melanoma, FTO is induced by metabolic starvation through autophagy, whereas knockdown of *ATG5* or *ATG7* in turn attenuates the expression of *FTO* [87]. In Leydig cells, human chorionic gonadotropin (HsCG) promotes the binding of the transcriptional factor C/EBPβ and TFEB to the promoter of *ALKBH5*, inducing its expression, whereas HsCG decreases *METTL14* expression, leading to the activation of AMPK-ULK1 axis and autophagy occurrence [83,88] (Figure 1).

## 3. Conclusions

Autophagy protects organisms against various pathologies, including pathogen infections, cancer, neurodegeneration, aging, and heart disease [1]. Many studies have unveiled that several transcription factors, including MIT/TFE, PPARα, ATF4, E2F1, C/EBPβ, FOXO, NF-κB, E93, STAT, and p53, are critical for autophagy induction in response to various upstream signal cascades [13,16,23,33,70]. TFEB acts as a master regulator of lysosomal biogenesis and autophagic flux with a dual role in nutrient deprivation and tumorigenesis [15]. Under nutrient-rich conditions, mTOR phosphorylates TFEB at the lysosomal surface, causing the retention of TFEB in the cytosol in mammals [89]. The removal of *MiT/TFE* factors from inhibition by mTORC1 promotes autophagy and lysosomal catabolism to maintain intracellular amino acids, playing the pro-survival role of cells in pancreatic ductal adenocarcinoma [90]. However, TFEB also acts as a negative regulator of autophagy since Rac1 selectively interacts with phosphorylated TFEB, preventing nuclear translocation of TFEB and thus inhibiting autophagy in HEK293 cells. Thus, the overexpression of the dephosphorylated form of TFEB delays tumor growth driven by Rac1, showing a positive activation of the Rac1-TFEB axis in tumorigenesis [91].

NF-κB is an important regulator of cellular immunity that is involved in the control of autophagy. NF-κB serves as a transcription factor with a dual effect on autophagy in different species. In the fly brain, NF-κB induces the expression of *Atg5* and *Atg7*, and thus triggers autophagy activation against infection with Zika virus [27]. However, TNFα upregulates mTOR activity in an NF-κB-dependent manner and inhibits autophagy in the human breast cancer MCF7 cell line [92]. FOXO3 coordinately activates protein degradation through the autophagic and proteasomal pathways in atrophying muscle cells; autophagy also helps protect cells by enhancing their capacity to destroy toxic protein aggregates. Therefore, activation of FOXO3, similarly to rapamycin treatment, stimulates autophagy and helps cells withstand the threat [16]. In summary, the molecular mechanisms of transcription factors in regulating autophagy are complex and usually condition specific.

An increasing number of non-coding RNAs are reported to regulate *Atg* genes [42]. The expression of genes encoding core autophagy-related proteins is regulated by RNA-binding proteins (RBPs) and certain non-coding RNAs, enriching the regulatory mechanisms of autophagy by epigenetics [93]. All microRNAs directly regulate *Atg* gene expression to affect autophagy, whereas in a series of tumors circRNAs serve as sponges of autophagy-related miRNAs to regulate autophagy [94]. LncRNAs directly regulate the expression of miRNAs through microRNA recognition elements (MREs) in lncRNA [95]. In addition, lncRNAs regulate the expression of *Atg* genes through competitive binding to microRNAs [70]. Information on microRNAs and lncRNAs that mediate autophagy can provide a starting platform to develop therapeutic strategies for cancer and neurodegenerative diseases [92]. Recently, a novel ncRNA regulator, called vault RNA (vtRNA), was reported to directly bind to autophagy receptor p62/SQSTM1 and change its function, acting as a riboregulator of key cellular processes such as autophagy [96]. The novel functions of microRNAs, circRNAs, and lncRNA, as well as their cooperative mechanisms in regulating autophagy, are worthy of further investigation.

Modifications of histone proteins and DNA methylation are two common epigenetic regulatory mechanisms of gene expression [97]. Recently, RNA modifications, such as RNA methylation, have attracted great attention. mRNA methylation occurs during pathophysiological processes of cell death and survival [79]. In particular, METTL3-mediated m^6^A methylation modification plays a critical role in autophagy and drug resistance of tumors [81]. However, the regulation of methyltransferases, e.g., METTL3, on autophagy are inconsistent in different cell types or physiological conditions, and the precise mechanism of mRNA methylation in regulating autophagy still needs further investigation [81]. m^6^A modification is a novel process able to regulate autophagy. Whether other RNA modifications are involved in this scenario is still unknown and deserves further research.

## Figures and Tables

**Table 1:** Transcription factors and their function in autophagy.

Transcription Factor	Function
Leucine zipper transcription factors (MiT/TFE)	MiT/TFE recognize promoters of lysosomal and *Atg* genes and represent transcriptional controllers of lysosomal biogenesis and autophagy [4,13].
Nuclear receptors PPARα and FXR	PPARα and FXR oppositely control the expression of *Atg7, Beclin1, Bnip3*, and *LC3* and autophagic vesicle formation [6].
Small heterodimer partner (SHP)	SHP decreases mRNA levels of *Atg* genes and inhibits autophagy [21].
Transcription factors FOXO/FOXA	Activation of FOXO/FOXA induces the expression of multiple *Atg* genes and lysosomal genes [17,32].
CCAAT/enhancer binding protein beta (C/EBPβ)	C/EBPβ targets key *Atg* genes and induces the expression of *Atg* genes [23,33].
Activating transcription factor 4(ATF4)	ATF4 is involved in the cellular stress response and autophagosome formation [5,34].
Nuclear factor-kappa B (NF-κB)	NF-κB activates the expression of *Atg* genes and induces autophagy [28,35].
Zinc-finger-family DNA-binding protein, ZKSCAN3	ZKSCAN3 decreases mRNA levels of *Atg* genes and inhibits autophagy [16].
Tumor suppressor p53	In the nucleus, P53 transactivates *Atg* genes and induces autophagy by inhibiting mTOR; in the cytoplasm, P53 suppresses autophagy [36,37].
Signal transducer and activator of transcription (STAT)	STAT3 phosphorylation upregulates *BNIP3* expression; STAT1 suppresses the expression of *Atg* genes [38,39].
Transcription factor E2F	Activation of E2F1 upregulates the expression of *Atg* genes [24].
TGA9 (TGACG motif-binding protein 9)	TGA9 activates autophagy by upregulating the expression of *Atg* genes [31].
E93	Knockdown of *E93* reduces the expression of several *Atg* genes in *B. mori* [40].
EcR-USP	20E-EcR-USP upregulates the transcription of *Atg* genes to induce autophagy [11].

**Table 2:** Non-coding RNAs involved in autophagy.

Non-Coding RNAs	Target Genes	Species	Impact on Autophagy
*miR30b*	*Atg12*, *Beclin-1*	*Helicobacter* *pylori*	↓[58]
*miR-17*	*Ulk1*	*Mouse*	↓[59]
*miR-30a*	*Beclin1, Atg12, Atg5*	*Mouse*	↓[44,60]
*miR-188-3p*	*Atg7*	*Mouse*	↓[61]
*miR-93, miR106b, miR142-3p*	*ULK1, ATG16L*	*Human*	↓[44,62,63]
*miR-101*	*ATG4D, LC3*	*Human*	↓[43,44]
*miR-155*	*ATG3*	*Human*	↓[64]
*miR-214-3p*	*ATG5, ATG12*	*Human*	↓[65]
*miR-216b*	*BECLIN1*	*Human*	↓[66]
*miR-103a-3p*	*ATG5*	*Human*	↓[45]
*miR-183, miR-204*	*LC3B1/LC3-II*	*Human*	↓[44,67]
*miR-83, miR-29*	*atg-4.2 / ATG4D, ATG9a*	*Caenorhabditis elegans/Human*	↓[8,46]
*miR-34*	*Atg9a/ATG9a*	*Caenorhabditis elegans/Human*	↓[47]
*miR-4459*	*ATG13*	*Human*	↓[68]
*miR-23b*	*ATG12*	*Human*	↓[69]
*miR-19a*	*BECLIN1, LC3*	*Human*	↓[70]
*miR-376b*	*ATG4C, BECLIN1*	*Human*	↓[44]
*miR-15a, miR-16*	*Rictor (mTORC1)*	*Human*	↑[71]
*circNF1-419*	*Dynamin-1*	*Mouse*	↑[50]
*circHIPK2*	*ATG5, BECLIN1-1*	*Human*	↑[72]
*circPABPN1*	*ATG16l1*	*Human*	↓[51]
*lncRNA APF*	*Atg7*	*Mouse*	↑[61]
*lncRNA NEAT1, lncRNA XIST*	*Atg9a*	*Mouse*	↑[8,53]
*lncRNA HAGLROS*	*PI3K-AKT-NF-κB*	*Human*	↑[73]
*lncRNA TGFB2-OT1*	*ATG3, ATG7, ATG13*	*Human*	↑[74]
*lncRNA CA7-4*	*AMPK*	*Human*	↑[75]
*lncRNA GBCDRlnc1*	*BECLIN1, ATG5, ATG12*	*Human*	↑[54,55]
*lncRNA MALAT1*	*Beclin1, LC3*	*Mouse*	↓[76]
*lncRNA LINC00470*	*BECLIN1, ATG3, ATG7*	*Human*	↓[77]
*lncRNA CTA*	*Unknown*	*Human*	↓[78]
*lncRNA HOTAIR*	*BECLIN1, LC3, ATG3, ATG7*	*Human*	↓[57]

Note: ↓: downregulation ↑: upregulation.
